# Persistence of immunity to SARS-CoV-2 over time in the ski resort Ischgl

**DOI:** 10.1016/j.ebiom.2021.103534

**Published:** 2021-08-12

**Authors:** Wegene Borena, Zoltán Bánki, Katie Bates, Hannes Winner, Lydia Riepler, Annika Rössler, Lisa Pipperger, Igor Theurl, Barbara Falkensammer, Hanno Ulmer, Andreas Walser, Daniel Pichler, Matthias Baumgartner, Sebastian Schönherr, Lukas Forer, Ludwig Knabl, Reinhard Würzner, Dorothee von Laer, Jörg Paetzold, Janine Kimpel

**Affiliations:** a Institute of Virology, Department of Hygiene, Microbiology and Public Health, Medical University of Innsbruck, Peter-Mayr-Str. 4b, Innsbruck 6020, Austria; b Department of Medical Statistics, Informatics and Health Economics, Medical University of Innsbruck, Austria; c University of Salzburg, Department of Economics, Residenzplatz 9, Salzburg A-5010, Austria; d Labor Dr. Theurl, Franz-Fischerstr.7b, Innsbruck, Austria; e Walser's surgery, Ischgl, Austria; f Department of Hygiene, Microbiology and Public Health, Medical University of Innsbruck, Innsbruck 6020, Austria; g Institute of Genetic Epidemiology, Medical University of Innsbruck, Austria

**Keywords:** SARS-CoV-2, Antibody response, T cell response, Persistence of immunity, Seroprevalence, Virus transmission

## Abstract

*Background* In early March 2020, a SARS-CoV-2 outbreak in the ski resort Ischgl in Austria triggered the spread of SARS-CoV-2 throughout Austria and Northern Europe. In a previous study, we found that the seroprevalence in the adult population of Ischgl had reached 45% by the end of April, representing an exceptionally high level of local seropositivity in Europe. We performed a follow-up study in Ischgl, which is the first to show persistence of immunity and protection against SARS-CoV-2 and some of its variants at a community level.

*Methods* Of the 1259 adults that participated in the baseline study, 801 have been included in the follow-up in November 2020. The study involved the analysis of binding and neutralizing antibodies and T cell responses. In addition, the incidence of SARS-CoV-2 and its variants in Ischgl was compared to the incidence in similar municipalities in Tyrol until April 2021.

*Findings* For the 801 individuals that participated in both studies, the seroprevalence declined from 51.4% (95% confidence interval (CI) 47.9–54.9) to 45.4% (95% CI 42.0–49.0). Median antibody concentrations dropped considerably (5.345, 95% CI 4.833 – 6.123 to 2.298, 95% CI 2.141 – 2.527) but antibody avidity increased (17.02, 95% CI 16.49 – 17.94 to 42.46, 95% CI 41.06 – 46.26). Only one person had lost detectable antibodies and T cell responses. In parallel to this persistent immunity, we observed that Ischgl was relatively spared, compared to similar municipalities, from the prominent second COVID-19 wave that hit Austria in November 2020. In addition, we used sequencing data to show that the local immunity acquired from wild-type infections also helped to curb infections from variants of SARS-CoV-2 which spread in Austria since January 2021.

*Interpretation* The relatively high level of seroprevalence (40–45%) in Ischgl persisted and might have been associated with the observed protection of Ischgl residents against virus infection during the second COVID-19 wave as well as against variant spread in 2021.

*Funding* Funding was provided by the government of Tyrol and the FWF Austrian Science Fund.

**Research in context**

**Evidence before this study**

In patient cohorts, several studies have recently shown persistence of immunity against SARS-CoV-2 using laboratory surrogate parameters such as antibodies and T cell responses, for up to 8 months.

**Added value of this study**

However, this is, to our knowledge, the first study at the community-level, where we show that the persistence of the laboratory markers of immunity was concomitant with reduced virus transmission within a population, for the wild-type virus (D614G) as well as for the B.1.1.7 variant.

**Implications of all the available evidence**

Our findings suggest that seropositivity levels of around 40–45% can persist in a community for over 8 months, which in turn may have helped to reduce the spread of both wild-type and B.1.1.7 transmission at the local level.

Alt-text: Unlabelled box

## 1 Introduction

Early on in the COVID-19 pandemic, it had already become clear that individuals previously infected with SARS-CoV-2 virus are immune to re-infection. Consequently, well-documented re-infections have been extremely rare to date, one year after the pandemic emerged [[1], [2], [3], [4], [5]]. However, it is still controversial how long this immunity will last. While some studies report a rapid loss of antibodies to SARS-CoV-2 within 2–4 months [6,7], especially in individuals after mild or asymptomatic infections, other studies have shown persistence of antibodies for several months, although levels decline over time [[8], [[9]], [10]]. In addition, recent studies have shown that T cell responses can still be detected 5–8 months after infection and most individuals after mild and asymptomatic infection retain at least T cell responses or neutralizing antibodies for up to 16–18 weeks [[11], [12], [13], [14]]. However, the question remains whether the long-term persistence of antibodies and T-cell responses detected in the laboratory after a local outbreak indeed is associated with effective community protection against SARS-CoV-2 infection and thus contributes to an increased immunity in the population.

In late April 2020, we studied the seroprevalence for SARS-CoV-2 in Ischgl, a popular ski resort in the Tyrolean Alps [15]. Ischgl was hit hard in March 2020 by the COVID-19 pandemic, and from Ischgl, the virus spread world-wide, mainly to Northern Europe and to the US [[16], [17], [18]]. A total of 1473 individuals, including 214 children, participated in our April study, corresponding to around 80% of the individuals living in Ischgl (residents and seasonal workers) at that time. We found that by the end of April 42% of the local population (45% of the adult population) had become seropositive [15]. This was one of the highest regional seroprevalence levels reported for spring 2020 and most of these seropositive individuals had been infected in March with a drastic decline of new infections in April.

In this paper, we report the results of a follow-up study (Ischgl 2) performed in the first week of November 2020, among adults, 6.5 months after the first study and up to 8 months after the first infection wave in Ischgl. First, we found that T-cell and antibody responses could still be detected in most individuals that had recovered from infection, and that Ischgl was relatively spared from the second SARS-CoV-2 wave that hit Austria in November 2020, compared to similar municipalities. Second, we showed that community protection also worked against some of the variants of SARS-CoV-2 and that this protection persisted until April 2021.

## 2 Methods

### 2.1 Ethics, study population, study design and recruitment

The ethical committee (EC) of the Medical University of Innsbruck approved the studies, baseline (Ischgl 1) with EC numbers: 1100/2020 and 1111/2020, which took place between April 21^st^ and 27^th^, 2020 and follow-up (Ischgl 2) with EC number: 1330/2020, which took place between November 2^nd^ and 8^th^, 2020. All inhabitants of Ischgl at the time point of the respective study were invited. After a patient briefing written informed consent was obtained from all participants. This cross-sectional epidemiological survey targeted all residents of Ischgl/Tyrol older than 18. At baseline, *n* = 1259 adults (82.4%) of all eligible adults (*n* = 1527) in Ischgl enrolled in the study [15]. Of the baseline sample, *n* = 813 (64.6%) entered the 6.5 months follow-up study. A total of *n* = 12 of these cases were excluded from analysis due to inconsistent age (*n* = 9), no questionnaire (*n* = 2) and no blood sample (*n* = 1). In total, *n* = 91 new participants entered the study at follow up; *n* = 12 of these were <18 years at baseline, *n* = 1 was excluded due to missing questionnaire data. Data have been collected with Askimed, a web-based eCRF system for data collection and management [19].

To study community spread, we analysed daily numbers of confirmed SARS-CoV-2 PCR-positive cases of all municipalities of Tyrol for 2020. In addition, we also received individual-level sequencing data with the number of B.1351 and B.1.1.7 cases from January until April 2021.

### 2.2 SARS-CoV-2 antibody tests

EDTA-plasma was analyzed for SARS-CoV-2-binding antibodies using four immunoassays. Samples were screened for anti-SARS-CoV-2-S1-protein IgA and IgG positivity by a commercially available anti-SARS-CoV-2-IgA and -IgG ELISA (Euroimmun, Lübeck, Germany), respectively, using the fully automated 4-plate benchtop instrument Immunomat™ (Virion/Serion, Würzburg, Germany). Results with respect to the obtained optical density (OD) values were interpreted according to the recommendations in the manufacturer's information. For both assays values >1.1 were considered positive. Borderline values (0.8–1.1) in the Euroimmun IgG ELISA were rated positive, for the Euroimmun IgA ELISA borderline values were rated as negative. Additionally, each plasma was tested for anti-SARS-CoV-2-N-protein IgG (anti-N IgG) with the Abbott SARS-CoV-2 IgG immunoassay on the ARCHITECT i2000SR system (Abbott, Illinois, USA), chemiluminescent microparticle immunoassay. Anti-N IgG was positive, if the obtained relative light unit (RLU) value corresponded to the manufacturer's recommendations (>1.4). Anti-N IgG was additionally quantified using the ElecsysAnti-SARS-CoV-2 (Roche Diagnostics, Indianapolis, USA) according to manufacturer's recommendations. The Roche assay detects antibodies, including IgG, against SARS-CoV-2 N protein using a double-antigen sandwich immunoassay design. This assay uses a cutoff index (COI), which is calculated using the standards provided by the manufacturer. A COI of ≥1.0 was considered positive. We used assays for detection of binding antibodies against both, N and S, as we found analyzing samples from healthy blood donors from beginning of 2019 that individual sera either showed cross-reactivity in S- or N-immunoassays (manuscript in preparation). However, we did not find a sample that showed cross-reactivity in both assays. Therefore, combination of both assays should decrease the number of false positive sampled.

### 2.3 Neutralizing antibody-assay

Titers of SARS-CoV-2 neutralizing antibodies were determined using a replication defective vesicular stomatitis virus (VSV) pseudotyped with SARS-CoV-2 spike protein or replication competent SARS-CoV-2 (D614G (B.1.177); B.1.351: GISAID EPI_ISL_1123262) as described previously [20]. Shortly, VSVΔG-GFP virus was produced on 293T cells stably expressing a C-terminally truncated version of SARS-CoV-2 spike (Wuhan variant, cells produced in-house as described in [20]). Four-fold serial dilutions of heat-inactivated plasma were pre-incubated with virus for 1 h at 37 °C and subsequently used to infect 293T-ACE2 cells (cells produced in-house as described in [20]) seeded the previous day. Approximately 16 h after infection, plates were analyzed in an ImmunoSpot® S5 analyzer (C.T.L. Europe, Bonn, Germany) and the number of GFP positive cells was counted. The last plasma dilution that resulted in a 50% reduction of GFP positive cells compared to virus only wells was considered as 50% neutralization titer. Titers of ≤1:4 were considered as negative, titers of ≥1:16 as positive. For the replication competent SARS-CoV-2, virus was pre-incubated with plasma dilutions and subsequently used to infect Vero-TMPRSS2 cells [21]. Ten hours after infection, cells were fixed, stained using the serum of a convalescent patient and the number of infected cells was counted using an ImmunoSpot S6 Ultra-V reader and CTL analyzer *BioSpot® 5.0* software (CTL Europe GmbH, Bonn, Germany). 50% neutralization titers were calculated using a nonlinear regression as described previously [22]. Titers ≤1:4 were considered as negative.

### 2.4 Defining seroprevalence and serostatus

**Fig. 1:**
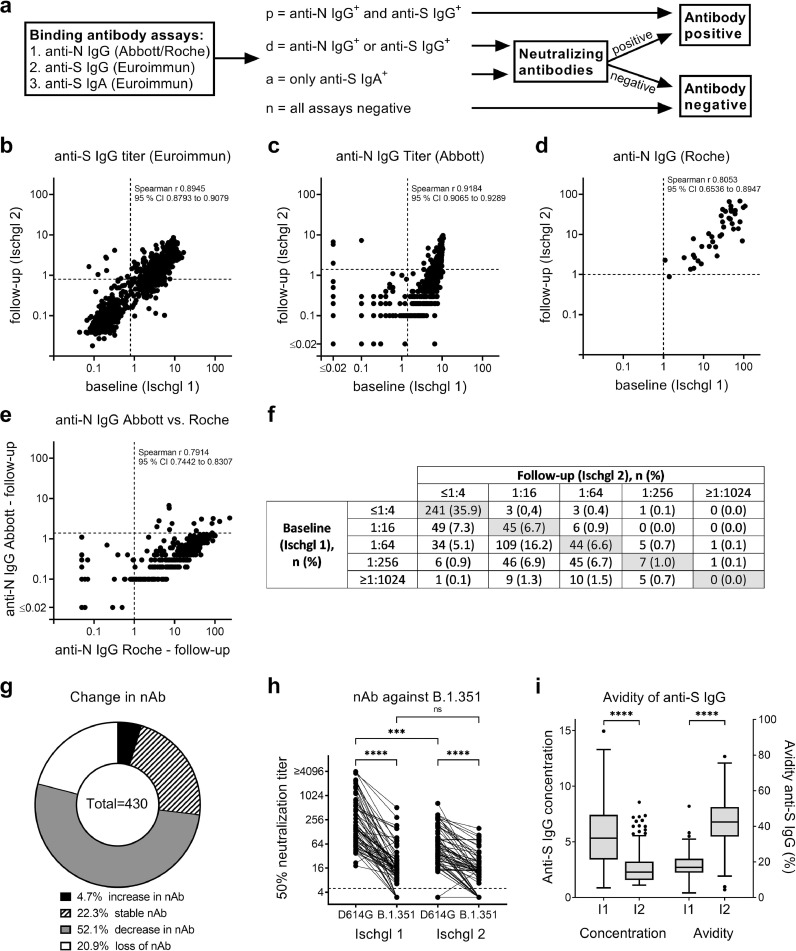
*Changes in SARS-CoV-2-specific antibodies.* (a) Antibodies were analyzed at baseline (Ischgl 1) and after 6.5 months follow up (Ischgl 2) using four immunoassays for binding antibodies and a neutralization assay. (b) Titers of anti-S IgG using Euroimmun ELISA. OD > 0.8 was counted as positive (dotted line), *n* = 801. (c) Titers of anti-N IgG using Abbott immunoassay. OD > 1.4 was counted as positive (dotted line), *n* = 801. (d) Titers of anti-N IgG using Roche immunoassay. OD > 1 was counted as positive (dotted line), *n* = 40 pairs of samples were analyzed. (e) Correlation of anti-N IgG determined via Abbott versus Roche assay at follow-up, *n* = 309. (b-e) Spearman r and 95% CI are depicted. (f) Titers of neutralizing antibodies. Titers ≤1:4 were counted as negative, *n* = 671 sample pairs were analyzed. (g) Change of neutralizing antibody titers between baseline and follow-up was analyzed for samples from f with positive (≥1:16) neutralizing antibody titers either in baseline or follow-up study. (h) Neutralizing antibody titers against wild-type (D614G) and B.1.351 were determined at baseline (Ischgl 1) and follow-up (Ischgl 2) using replication competent SARS-CoV-2 isolates (*n* = 66). Titers ≤1:4 were considered negative (dotted line). Statistics were determined using One-Way ANOVA (Friedman test with Dunn's multiple comparison), ns = non-significant, *** *p* < 0.001, **** *p* < 0.0001. (i) Avidity of anti-S IgG was analyzed at baseline and follow-up for all samples with positive anti-S IgG titers at both time points, n = *n* = 218. Temporal trend in the median (IQR) concentration of anti-SARS-CoV-2 IgG antibodies as compared to the median binding affinities (avidities) of the antibodies (%). The lower and upper bars represent the minimum and maximum values, respectively. Dots represent outliers. Statistics were determined using Students t-test for paired samples, **** *p* < 0.0001.

Plasma samples were analyzed according to the scheme in Fig. 1a. The serostatus of the samples was defined as p, d, a or n depending on the binding antibody assays:

*p* = positive = anti-S IgG^+^ AND anti-N IgG^+^ (either Roche or Abbott assay positive)
*d* = discordant = anti-S IgG^+^ OR anti-N IgG^+^ (either Roche or Abbott assay)
*a* = only IgA = only anti-S IgA^+^ but anti-S IgG^−^ AND anti-N IgG^−^
*n* = negative = anti-S IgG^−^ AND anti-N IgG^−^ AND anti-S IgA^−^

To calculate the seroprevalence, all individuals with serostatus *p* were considered as seropositive. For serostatus d and a, individuals were considered as seropositive when they had neutralizing antibodies ≥1:16.

### 2.5 Avidity of SARS-CoV-2-specific anti-S IgG

A kit was used according to manufactures instructions to determine of the avidity of anti-S antibodies (Euroimmun AG, Lübeck, Germany, REF: EI 2606–9601 G). Shortly, plasma samples, in a 1:101 dilution, were added to microplate wells coated with an S1-domain of the spike protein of SARS-CoV-2 and incubated in duplicates for 60 min at 37 °C. After three washing steps, the pairs of samples were treated either with 200 µl urea or 200 µl PBS and were further incubated for 10 min. Plates were washed again. For the detection of remaining antibody, 100 µl of peroxidase-conjugated anti-human IgG were added, incubated for another 30 min at 37 °C and plates were subsequently washed. For the colorimetric signal detection TMB substrate was used. Reaction was stopped after a 30 min incubation at room temperature and plates were measured at wavelengths of 450 nm (signal) and 620 nm (reference wavelength for background subtraction) using a Tecan Sunrise Reader (Grödig, Austria). The relative avidity index (RAI) for each sample corresponded the ratio of the optical densities (ODs) with and without urea incubation.

### 2.6 Analysis of SARS-CoV-2-specific T cell responses

For details regarding T cell isolation and analysis see supplementary methods.

To study SARS-CoV-2 specific T cell responses Prot_S, Prot_M and Prot_N SARS-CoV-2 PepTivator® (Miltenyi Biotech, Bergisch-Gladbach, Germany) peptide pools consisting mainly of 15-mer sequences with 11 amino acids overlap covering the immunodominant sequence domains of the surface (or spike) glycoprotein (pepS), and the complete sequence of the membrane glycoprotein (pepM) as well as the nucleocapsid phosphoprotein (pepN) were used. As positive control PepTivator® CEF MHC Class 1 Plus (pepCEF) was used, consisting of 32 MHC class 1-specific peptides of 8–12 amino acids in length derived from human cytomegalovirus (HCMV), Epstein-Barr virus (EBV), and influenza A virus. Alternatively, cells were stimulated with Phytohaemagglutinin (PHA, Sigma) at a concentration of 10 µg/ml.

To expand SARS-CoV-2 reactive T cells, 2 × 10^6^ PBMCs in 500 μL of RPMI medium supplemented with 2% human AB serum were stimulated with 1 µg/ml pepS, pepM or pepN peptide pools in the presence of 20 U/ml interleukine-2 (IL-2). As negative control, cells were cultured with IL-2 alone. PepTivator pepCEF at 1 µg/ml in the presence of IL-2 was used as positive control. At day 3, 500 μL fresh RPMI medium supplemented with 2% human AB serum and 40 U/ml IL-2 was added. At day 7, cells were harvested and counted. A total of 1 × 10^5^ cells were re-stimulated with or without 1 µg/ml pepS, pepM or pepN peptide pools. As positive controls, cells were stimulated with 10 µg/ml PHA or re-stimulated with PepTivator pepCEF at 1 µg/ml. Specific T cell responses were analyzed in an IFNγ enzyme-linked immunospot (ELISPOT) or by intracellular (IC) cytokine and cell surface staining (Supplementary Methods).

### 2.7 Statistical analysis

Statistical analyses concerning the community spread in Ischgl vs. control municipalities (based on the daily numbers of confirmed SARS-CoV-2 cases as well as the sequencing data) were performed in Stata 16.1 (StataCorp. 2019. Stata Statistical Software: Release 16. College Station, TX: StataCorp LLC). Statistical analysis of antibody and T cell responses has been performed with GraphPad Prism version 9 (GraphPad Software, Inc., La Jolla, CA, USA). Demographic characteristics were tabulated using descriptive statistics including the calculation of means ± standard deviations (or median and interquartile range (IQR)) for continuous measures and numbers (%) for categorical measures. 95% confidence intervals for binomial proportions, including crude prevalence estimates of seroprevalence, were calculated using the Clopper-Pearson estimation method. Cross-sectional seroprevalence was calculated in November for the whole sample, seroprevalence at baseline and follow-up was also calculated for individuals for whom data was available for both.

Quantitative variables were compared across groups using parametric (ANOVA) and non-parametric methods (Mann-Whitney U-test, Kruskal-Wallis test). Differences in quantitative variables at baseline and follow-up were compared using parametric (Student's t-test for paired samples, repeated measures ANOVA) and non-parametric (Wilcoxon signed-rank test) tests. Associations between categorical variables were tested using χ² test, with Fisher's exact test where appropriate. A two-tailed value of *p* < 0.05 was considered statistically significant for all comparisons.

### 2.8 SARS-CoV-2 transmission in Ischgl in 2020

We compared daily numbers of confirmed SARS-CoV-2 PCR-positive cases of Ischgl with 13 control municipalities. We selected 5% of all municipalities in the federal state of Tyrol (279 in total) that are most similar to Ischgl in terms of the Mahalanobis distance in covariate space. The covariates used to calculate the Mahalanobis distance were population size, settlement area per square km, share of female population, share of people under 16 years, share of people older than 65 years, number of commuters, share of people with tertiary education, and hotel bed capacity. The matching approach selected control group municipalities which are most similar to Ischgl. The selected control municipalities were Eben am Achensee, Ellmau, Fiss, Gerlos, Lermoos, Leutasch, Mayrhofen, Nauders, Neustift im Stubaital, Seefeld in Tirol, Serfaus, Sölden, Tux. These are all very tourism-intense holiday towns in Tyrol, and very comparable to Ischgl in terms of the covariates used. All calculations were executed with the software R (version 4.03), and the R-package Synth [8].

### 2.9 Role of Funders

The study has been supported by the state of Tyrol. KB has been supported by a FWF Austrian Science Fund Lise Meitner Award [M-3069-B]. The funders had no role in study design, data collection, data analysis, interpretation, writing the manuscript or the decision to submit it for publication. The authors have not been paid by a pharmaceutical company or other agency to write this article. The corresponding authors had access to all the data of the study and had final responsibility for the decision to submit for publication.

## 3 Results

### 3.1 Study population

In the first week of November 2020, 6.5 months after our first seroprevalence study in the ski-resort Ischgl (baseline study = Ischgl 1), we again invited all adult Ischgl residents to participate in a study on the level of local immunity to SARS-CoV-2 (follow-up study = Ischgl 2). In April 2020, an estimated 1527 adults lived in Ischgl, of these 1259 participated in Ischgl 1 (82.4%). In November, an estimated 1304 adults were living in Ischgl, this decrease reflects the absence of seasonal workers. Of these, 904 adults participated in November (69.3% participation rate). Of the 1259 study participants at baseline, 801 individuals participated again in November and were included in the analysis in the follow-up study presented here (63.6%, Supplementary Fig. 1). Age distribution was similar in the adult population in both studies, while there was a slight bias towards female participants in Ischgl 2 (54.9%) relative to Ischgl 1 (51.9%) (Supplementary Table 1).

### 3.2 Antibodies to SARS-CoV-2 were still detectable although the levels declined

The testing strategy for antibodies to SARS-CoV-2 is depicted in Fig. 1a. IgG antibodies against spike (S) and nucleoprotein (N) were determined using commercially available immunoassays. We observed that many individuals had lost anti-N IgG antibodies in the Abbott anti-N IgG-Abbott test in Ischgl 2, but turned out to still have anti-N IgG antibodies in the Roche anti-N IgG-Roche assay (Supplementary Table 2). For individuals negative in all three assays, anti-S IgA antibodies were determined using an anti-SARS-CoV-2-IgA ELISA (Euroimmun, Lübeck, Germany). We defined the serostatus groups p (positive), d (discordant), a (IgA to S protein only), and n (negative in all test), based on the outcome of the commercial antibody assays as explained in Material and Methods and in Fig. 1a.

For all individuals with serostatus d and a, neutralizing antibodies were determined. The following individuals were considered seropositive: p (anti-S IgG and anti-N IgG positive), d (discordant) plus neutralizing antibody positive; a (anti-S IgA only) plus neutralizing antibody positive. We have previously described a seroprevalence of 45.0% (95% CI 42.2 – 47.8) for all adult participants at Ischgl 1 (*n* = 1259) [15]. For the follow-up study, there was a slight bias for participation of baseline study seropositive individuals, while fewer negative individuals returned. This resulted in a baseline study seroprevalence of the subpopulation included in both studies (*n* = *n* = 801) of 51.4% (95% CI 47.9–54.9). Here, we determined the seroprevalence for all adult participants at Ischgl 2 (*n* = 891) with 44.7% (95% CI 41.4 - 48.0). For individuals that participated in both studies (*n* = 801), the level of seropositivity declined between both studies to 45.4% (95% CI 42.0–49.0) in Ischgl 2 (Supplementary Table 3). From the 90 individuals that only participated in Ischgl 2 and not in Ischgl 1, 37.8% (95% CI 27.8–48.6) were seropositive in Ischgl 2 (Supplementary Table 3). The individuals that only participated in Ischgl 2 were not otherwise included in the following analysis.

`Median antibody levels to SARS-CoV-2 significantly declined in each assay - by 50% in the anti-S IgG assay (*n* = 801, Z =22.0, p< 0.001, Wilcoxon signed-rank test), 88.9% in the anti-N-IgG-Abbott (*n* = 801, Z = 19.4, *p* < 0.001, Wilcoxon signed-rank test) and 58.6% in the anti-N-IgG-Roche assay (*n* = 40, Z = -4.4, p< 0.001, Wilcoxon signed-rank test) (Fig. 1 b–d and Supplementary Table 2). The higher decline of anti-N antibodies in the Abbott assay compared to the Roche assay indicates a higher sensitivity of the Roche assay especially for detection of antibodies late after infection, which can also be seen when directly comparing the same samples from Ischgl 2 in both assays (Fig. 1e). In several individuals, antibodies were lost for one of the antigens switching from positive “p” to discordant “d” (p-d) (Table 1). The majority of these individuals with serostatus p-d retained antibodies to N but lost anti-S binding antibodies (81 out of 82 p-d individuals). Interestingly, ∼3/4 of these p-n individuals that lost binding antibodies to S retained neutralizing antibodies. This might be explained by conformation dependent neutralizing antibodies that are not recognized in the ELISA. Only 4 of 364 (1.1%, 95% CI 0.–2.8) individuals with serostatus p (positive for anti-S and anti-N antibodies) in Ischgl 1 were tested negative in all commercial antibody test kits in Ischgl 2 (group p-n, Table 1). Interestingly, 3 of the 4 individuals in the p-n group (75%) still retained neutralizing antibodies (Table 1). Of the 8 sera that were only positive for IgA antibodies to S protein in Ischgl 2, 2 had neutralizing antibodies (Table 1).

**Table 1:** Serostatus at baseline (Ischgl 1) and follow-up (Ischgl 2)

		**Serostatus at follow-up (Ischgl 2), *n* (%), NT^+^/total NT assays**				
		Positive - *p*	Discordant - *d*	IgA only - *a*	Negative - *n*	Total
**Serostatus at baseline (Ischgl 1), *n* (%), NT^pos^/total NT assays**	Positive - *p*	276 (34.5) 244 NT^pos^/276	82 (10.2) 63 NT^pos^/82	2 (0.2) 2 NT^pos^/2	4 (0.5) 3 NT^pos^/4	364
	Discordant - *d*	4 (0.5) 2 NT^pos^/4	30 (3.7) 13 NT^pos^/30	1 (0.1) 0 NT^pos^/1	8 (1.0) 2 NT^pos^/7	43
	IgA only - *a*	0 (0.0)	1 (0.1) 0 NT^pos^/1	3 (0.4) 0 NT^pos^/3	7 (0.9) 0 NT^pos^/7	11
	Negative - *n*	4 (0.5) 3 NT^pos^/4	5 (0.6) 2 NT^pos^/5	2 (0.2) 0 NT^pos^/2	372 (46.4) 6 NT^pos^/246	383
	Total	284 (35.5)	118 (14.7)	8 (1.0)	391 (48.8)	801

NT^pos^ (Neutralizing antibody titer positive) = titer ≥1:16

Also, the neutralizing antibody titers declined in most individuals that were neutralizing antibodies positive in Ischgl 1, and 90 of 430 (20.9%, 95% CI 17.2 - 25.1) individuals completely lost neutralization capacity in our assay (Fig. 1f,g). Interestingly, of these 90 individuals that lost neutralizing antibodies in Ischgl 2, 1 had IgA to S, 33 had IgG antibodies to either N or S (3 S+/N- and 30 S-/N+) and 32 had antibodies to both antigens in the commercial antibody assays. The decline of neutralizing antibodies between both studies was also observed in a second neutralization assay using replication competent primary SARS-CoV-2 isolates, where we analyzed a subpopulation of 66 patients for wild-type virus (D614G) and B.1.351 variant (Fig. 1h). Neutralizing antibody titers against the B.1.351 variant were significantly lower compared to titers against wild-type virus in both studies. However, there was no significant decline of the level of B.1.351 cross-neutralizing antibodies between Ischgl 1 and Ischgl 2.

We then studied the avidity of the antibodies in individuals that were anti-S IgG positive in Ischgl 1 and 2 (Fig. 1i). While the antibody titers to S had declined over time, the avidity significantly increased as a sign of antibody maturation. Thus, despite the lower concentration of antibodies in Ischgl 2, the antibodies that persisted show a higher binding strength and thereby most likely an improved functionality.

### 3.3 T cell response

**Fig. 2:**
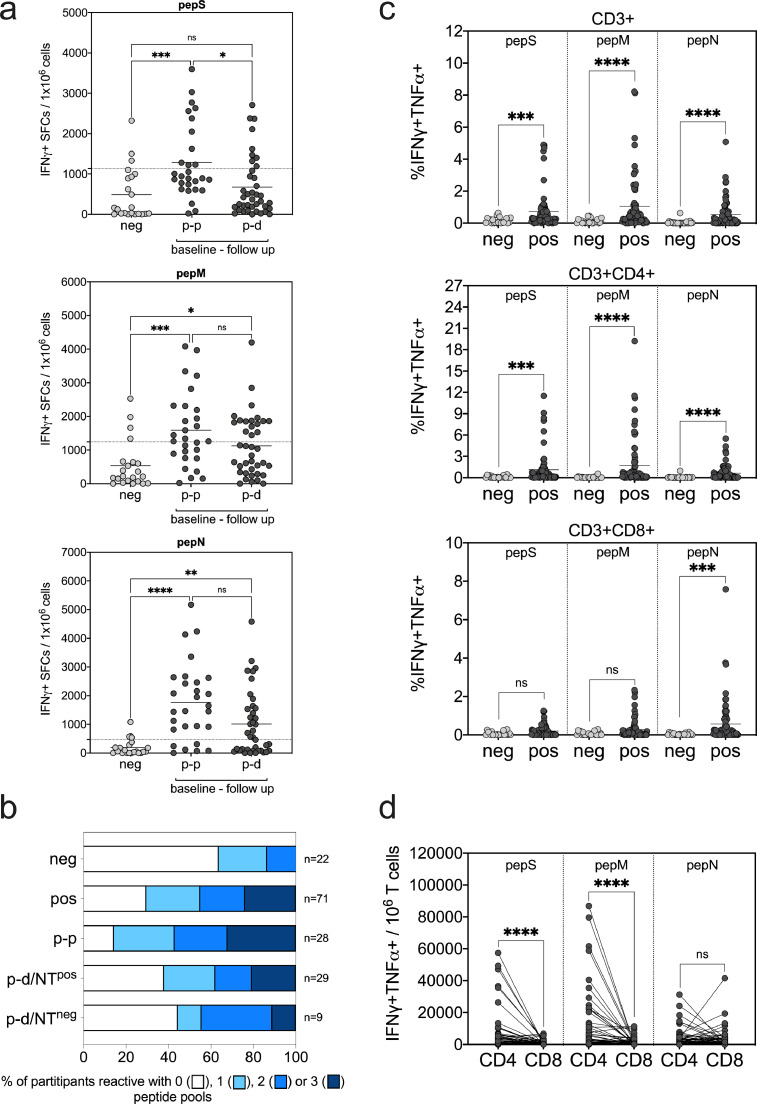
*T cell responses against SARS-CoV-2-derived peptide pools.* Analysis of SARS-CoV-2-specific T cells by (a,b) IFNγ ELISPOT assay and (c,d) IFNγ/TNFα intracellular cytokine staining (ICS) after a 7-day in vitro expansion followed by re-stimulation with pepS, pepM and pepN peptide pools. Values after peptide re-stimulation were normalized to non-stimulated samples in both ELISPOT and ICS. (a) IFNγ positive SFCs per 10^6^ cells are shown after pepS, pepM and pepN re-stimulation. Negative study group (neg, *n* = 22) is compared to baseline positive with a follow up serostatus of positive (p-p, *n* = 28) and discordant (p-d, *n* = 38). Dotted lines show the cut-off of the assay, above which T cell responses were declared reactive in the ELISPOT assay and are defined as the mean + 1 × SD of the negative group for the respective peptide pool. (b) Proportion of participants reactive with 0, 1, 2 or 3 peptide pools is shown in baseline antibody negative and baseline antibody positive study groups. Baseline antibody positive participants were additionally divided according to serostatus in follow-up study, p-p (baseline and follow-up positive), p-d/NT^pos^ (baseline positive, follow-up discordant but neutralizing antibody positive) and p-d/NT^neg^ (baseline positive, follow-up discordant but neutralizing antibody negative). (c) Percentage of IFNγ/TNFα-producing CD3+ total T cells, CD3+CD4+ helper T cells and CD3+CD8+ cytotoxic T cells after peptide re-stimulation are shown. After ICS living/singlet cells were gated for CD3+ total, CD3+CD4+ and CD3+CD8+ T cells and the indicated percentage depicts the frequency of IFNγ/TNFα double-positive cells in the respective population of the sample stimulated with the test peptide minus the frequency of the non-stimulated control. (d) Number of IFNγ/TNFα double-positive CD4+ and CD8+ cells per 10^6^ CD3+ total T cells were calculated and depicted. For (c) and (d), *n* = 22 (neg) and *n* = 71 (pos). Statistical analysis for data in (a) were done by ANOVA and significance were calculated by Kruskal-Wallis test followed by Dunn's multiple comparison, for data (c) and (d) significance were calculated by Mann-Whitney test (ns, not significant; *, **, *** and **** represent p values <0.05, <0.01, <0.001 and <0.0001, respectively).

In Ischgl 1, T cell response had not been analysed. To study if the decline in humoral immunity was compensated at least partially by T cell responses in Ischgl 2 participants, we analyzed the response to peptide pools derived from S, M and N protein of SARS-CoV-2 by ELISPOT (Fig. 2a,b) and intracellular cytokine staining (ICS) analysis (Fig. 2c,d). We analyzed a total of 71 antibody positive individuals from Ischgl 1, who remained positive (p-p, *n* = 28, all NT^pos^ (neutralizing antibody titer positive)), became discordant (p-d, *n* = 38, 29 NT^pos^/9 NT^neg^ (neutralizing antibody titer negative)), or IgA only (p-a, *n* = 1, NT^pos^) or became negative (p-n, *n* = 4, 3 NT^pos^/1 NT^neg^) in Ischgl 2. Individuals that were antibody negative in both studies (*n* = 22, all NT^neg^) served as a control group. As positive controls, we used pepCEF and PHA, and T cell response to these stimulations were comparable in all investigated groups (Supplementary Fig. 2).

Although approximately one third of individuals in the seronegative group showed T cell responses to at least one peptide pool (Table 2, Fig. 2b), the responses were significantly higher in baseline seropositive individuals (Fig. 2a–c). The responses to pepM and pepN pools were not significantly different between the different groups of positive individuals studied. The pepS pool response was not significantly difference between n-n and p-d groups, however the response in p-p group was found significantly higher compared to both n-n and p-d groups. The T cell response was primary elicited by CD4+ T cells and to a lesser extent by CD8+ T cells for the S and M protein peptide pools, while CD4+ and CD8+ T cells contributed equally to the response against the N peptide pool (Fig. 2c,d).

**Table 2:** Number of T cell reactive* and neutralizing study participants (*n* = 93)

Number of reactive peptide pools	baseline neg	baseline pos	baseline - follow up					
			p-p§	p-d (NT^pos^)	p-d (NT^neg^)	p-a§	p-n (NT^pos^)	p-n (NT^neg^)
0	14/22 (63.6)#	21/71 (29.6)	4/28 (14.3)	11/29 (37.9)	4/9 (44.4)	0/1 (0)	1/3 (33.3)	1/1 (100)
1	5/22 (22.7)	18/71 (25.4)	8/28 (28.6)	7/29 (24.1)	1/9 (11.1)	0/1 (0)	2/3 (66.6)	0/ 1 (0)
2	3/22 (13.6)	15/71 (21.1)	7/28 (25.0)	5/29 (17.2)	3/9 (33.3)	0/1 (0)	0/3 (0)	0/1 (0)
3	0/24 (0)	17/71 (23.9)	9/28 (32.1)	6/29 (20.7)	1/9 (11.1)	1/1 (100)	0/3 (0)	0/1 (0)

* IFNγ+ SFCs / 10^6^ cells > mean + 1 × SD of negative study participants

# number of reactive samples/number of total samples (% reactive)

§ baseline – follow up serostatus p-p: positive - positive, p-d: positive - discordant, p-a: positive - IgA only, p-n: positive – negative, NT^pos^: neutralizing antibody positive (titer ≥1:16) at follow-up; NT^neg^: neutralizing antibody negative (titer ≤1:4) at follow-up

When analyzing the number of reactive peptide pools, we found significant difference between baseline antibody negative and positive study groups (Table 2, *p* = 0.007, χ² test, with Fisher's exact test). When comparing baseline negative participants with baseline positives in the different subsets in the follow-up, the number of reactive peptide pools in the p-p group but not p-d/NT^pos^ and p-d/NT^neg^ groups was significantly higher than in the n-n group (p-p versus n-n p=0.001, p-d/NT^pos^ versus n-n p=0.091 and p-d/NT^neg^ versus n-n p=0.261, χ² test, with Fisher's exact test). Thus, T cell response in term of the number of reactive peptide pools correlated with declining serostatus with p-p > p-d/NT^pos^ > p-d/NT^neg^ (Table 2, Fig. 2b). Importantly, we found that of the 9 individuals that had lost binding antibodies partially and neutralizing antibodies completely (p-d/NT^neg^), 5 still had a detectable T cell response, thus those individuals could potentially still have some T cell-mediated immune protection. Of the four individuals in the p-n group that had lost all antibodies in the commercial assays, 3 still retained neutralizing antibodies, two of which had detectable T cell responses and only one individual had lost all binding and neutralizing antibodies and also showed no T cell responses (Table 2).

Overall, we found that antibody T cell responses or at least one of both were still detectable in most individuals that were positive in the first study during the follow-up study indicating that responses persisted for up to 8 months.

### 3.4 Virus transmission in Ischgl

Between Ischgl 1 and 2, 4 individuals seroconverted, of which one individual had been tested positive by PCR in October 2020. Two of the remaining 3 reported symptoms compatible with COVID-19 between the two studies (Supplementary Table 4).

We then investigated whether the high-level immunity in Ischgl, which was still detected in November, had limited virus transmission during the second wave of SARS-CoV-2, which hit Austria in autumn 2020. We compared daily numbers of confirmed SARS-CoV-2 PCR-positive cases of Ischgl with 13 control municipalities selected from Tyrol. Notice that information on the number of undertaken PCR-tests was not available at the municipality level. However, participation rates in a free and voluntary mass antigen test conducted in the province of Tyrol on the weekend of December 5^th^/ 6^th^, 2020 were very similar between Ischgl and the control municipalities (28 vs. 33%). This suggests that the willingness of Ischgl citizens to test did not differ compared to other municipalities.

**Fig. 3:**
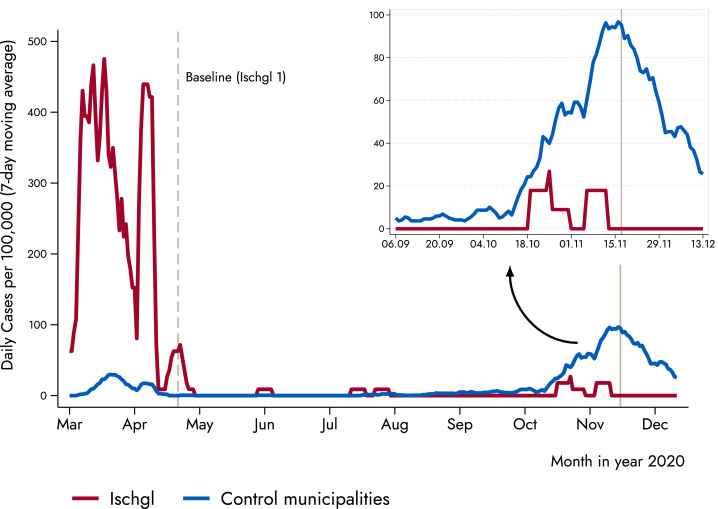
*Lower incidence of new infections in Ischgl compared to low-prevalence villages.* The figure displays the 7-day moving average of new cases between Ischgl and the control municipalities. Vertical solid line represents the second countrywide lockdown in autumn 2020, which took place in November 17 (a first and lighter lockdown took place on November 2). The control municipalities are Eben am Achensee, Ellmau, Fiss, Gerlos, Lermoos, Leutasch, Mayrhofen, Nauders, Neustift im Stubaital, Seefeld in Tirol, Serfaus, Sölden, and Tux.

Fig. 3 shows the 7-day moving average of daily new confirmed SARS-CoV-2 cases per 100,000 inhabitants for Ischgl and the control municipalities. Three things stick out: First, the figure confirms that Ischgl was severely hit during the first wave of the pandemic, with the daily number of cases being an order of magnitude larger compared to the control municipalities. Second, during the summer in both Ischgl and the control municipalities the number of cases converged to (almost) zero, as in most places in Europe. Third, with the start of the second wave in October 2020, the trend between the two groups sharply diverged, with the control municipalities showing a substantially larger number of confirmed cases (Fig. 3). The observed difference in confirmed SARS-CoV-2 PCR-positive cases between Ischgl and the control municipalities supports the hypothesis that the high seroprevalence of approximately 40–45% in Ischgl contributed to the containment of virus transmission.

### 3.5 Emergence of SARS-CoV-2 Variants-of-Concern and its effect on local transmission

In the end of 2020, first reports about the spread of two SARS-CoV-2 Variants-of-Concern (B.1.1.7 and B.1.351) in Europe surfaced (23]. By February 2021, one of the largest outbreaks of B.1.351 in Europe occurred in the state of Tyrol (24]. The government of Tyrol responded with strong disease control measures, including the sequencing of almost all SARS-CoV-2 PCR-positive cases of the region.

Overall, we observed 495 SARS-CoV-2 PCR-positive cases, with 10 of those in Ischgl and 485 in our control municipalities between December 23^rd^ and April 6^th^ (end of sample period). Sequencing these cases, we found that 222 were wild-type (6 Ischgl, 216 control municipalities), 110 the B.1.1.7 variant (4 Ischgl, 106 control municipalities) and 99 the B.1.351 variant (0 Ischgl, 99 control municipalities). Using this data, we first analysed whether the overall number of cases (B.1.351 + B.1.1.7 + wild-type) continued to be significantly larger in the control municipalities compared to Ischgl from January to April 2021. Specifically, we applied a two-sample t-test (with equal variances) to test for a significant difference in the 7-day moving average of daily new confirmed COVID-19 cases per 100,000 inhabitants between Ischgl and the control municipalities. We found the mean of the 7-day moving average of daily new cases to be 17.4 (95% CI 15.9–18.8) for the control municipalities, and 7.5 (95% CI 5.6–9.4) for Ischgl. The difference amounts to 9.9 and is statistically significant (*p* < 0.001, two-sample t-test). Applying the same two-sample t-test on B.1.1.7 cases only, we again found that Ischgl had a significantly lower number of cases compared to the control municipalities (*p* < 0.07). The mean of the 7-day moving average of daily new B.1.1.7 cases is 4.1 (95% CI 3.1–5.2) for the control municipalities, and 2.8 (95% CI 1.3– 4.3) for Ischgl. Regarding the wild-type, we observed a statistically significant difference of 4.4 (*p* < 0.001, two-sample t-test; Ischgl: 4.7, 95% CI 3.0 – 6.4; Control municipalities: 9.1, 95% CI 7.8 – 10.4). While we observed 99 B.1351 cases in our control municipalities between January and April 2021, we did not observe a single case of this variant in Ischgl. B.1351 was mostly found in the eastern districts of Tirol rather than in the west, where Ischgl is located.

These results suggest that Ischgl had relatively less spread of both the wild-type and the B.1.1.7 variant compared to the control municipalities. However, the two-sample t-test cannot tell us whether the wild-type and B.1.1.7 spread similarly within Ischgl. Recent research based on neutralization assays suggest that antibodies acquired from wild-type infections work similarly well against B.1.1.7 [25]. However, it remains an open question whether this also applies to the population-level immunity. Thus, we used our individual sequencing data to test more directly whether the B.1.1.7 variant spread differently to the wild-type in Ischgl. Specifically, we used all individuals tested SARS-CoV-2 PCR-positive from Ischgl and the control municipalities and constructed an indicator variable which equals 1 if the positive case was a B.1.1.7, and 0 if wild-type. We then regressed this dummy variable on the age of the individual (the only personal characteristic in our data), and a dummy variable which took a value of 1 when the individual is a resident of Ischgl, and 0 otherwise. The dummy variable for Ischgl turned out to be insignificant (-0.064; 95% CI -0.355–0.227). This indicates that while Ischgl had a significantly lower number of both wild-type and B.1.1.7 cases compared to the control municipalities, the likelihood to get infected with B.1.1.7 relative to wild-type is not statistically different for a person from Ischgl compared to a person from a control municipality. Hence, it seems that immunity acquired from wild-type infections curbed new infections from the wild-type and B.1.1.7 in a similar way. Our epidemiological results are in line with laboratory evidence, indicating that B1.1.7 is not more resistant to plasma from individuals who have recovered from COVID-19 wild-type.

## 4 Discussion

Our findings suggest that the immunity to SARS-CoV-2 on the community level can persist for at least eight months. We found that both antibodies and T cell responses persisted and that this might translate into a reduced local virus transmission.

Our study extends the previous research in three important ways: First, we confirm that immunity as defined by T cell and/or neutralizing antibody response lasts for up to several months and that T cell and antibody responses can be discordant [[8], [[9]], [10], [11], [12], [13], [14],^26^]. The majority of the 412 adults that were seropositive during the Ischgl 1 study in late April and analyzed again in early November, Ischgl 2, had been infected already in March during the first wave. Thus, our study represents one of the longest and largest follow-up studies published so far.

Second, since our study was performed in a population within a defined narrow geographical area, we were able to analyze the epidemiological transmission of SARS-CoV-2 within this population along with immunity patterns in the laboratory. We found that the incidence of SARS-CoV-2 in Ischgl was lower than in comparable municipalities during the second wave that hit Austria in November 2020. The reduced incidence in Ischgl relative to comparable municipalities (as well as to the rest of Austria) indicates that the level of immunity to SARS-CoV-2 helped to curb new infections in Ischgl.

Finally, our findings suggest that seropositivity levels of around 40-45% can persist in a community for over 8 months, which in turn may have helped to reduce the spread of both wild-type and B.1.1.7 transmission at the local level.

There are some limitations of our study. In the follow-up study relative more of the baseline study seropositives returned compared to the baseline study negatives. This might have introduced a bias in the seroprevalence calculation of the follow-up study. However, seroprevalence of the study participants that were new in the follow-up study was in a similar range. Additionally, T cells were only analyzed in the follow-up study; hence development of T cell responses is difficult to judge. Lastly, we cannot distinguish between the effects of the high seroprevalence and of non-pharmacological interventions on virus transmission in Ischgl as at the time of our follow-up study lockdown light measures were in place. However, the lockdown light measures were identical in Ischgl and the rest of Austria.

## 5 Contributors

WB, ZB, HW, RW, DvL, JP, JK designed experiments; ZB, LR, AR, LP, DP, MB performed experiments; WB, ZB, KB, HW, IT, BF, HU, LK, RW, DvL, JP, JK analyzed data; WB, KB, SS, LF designed questionnaire; AW the local general practitioner, supervised the study in Ischgl; WB, ZB, KB, HW, DvL, JP, JK had access to all data and reviewed them; HW, DvL, JP, JK wrote the paper; all authors read and approved the manuscript.

## 6 Data sharing statement

Upon publication of our study, the data and codes will be available from the corresponding authors upon receipt of a suitable request.

## Declaration of Competing Interest

IT has contracts with Kymab and Sierra Oncology and received consulting fees from Kymab. For the other authors, no conflicts of interests exist. The funders had no role in the design of the study; in the collection, analyses, or interpretation of data; in the writing of the manuscript, or in the decision to publish the results.

## Appendix Supplementary materials

Image, application suppl1
